# Supplementary material for: Comprehensive Annotation and Functional Exploration of MicroRNAs in Lettuce
Source: Front Plant Sci. 2021 Dec 24;12:781836. doi: 10.3389/fpls.2021.781836 (PMC8739914; doi:10.3389/fpls.2021.781836)
Supplement: Supplementary file 2 [file Data_Sheet_8.ZIP › GSM2230750.plot/Lsa-miRN1700_Lsat_1_v5_gn_6_60280.1_40_TPlot.pdf]

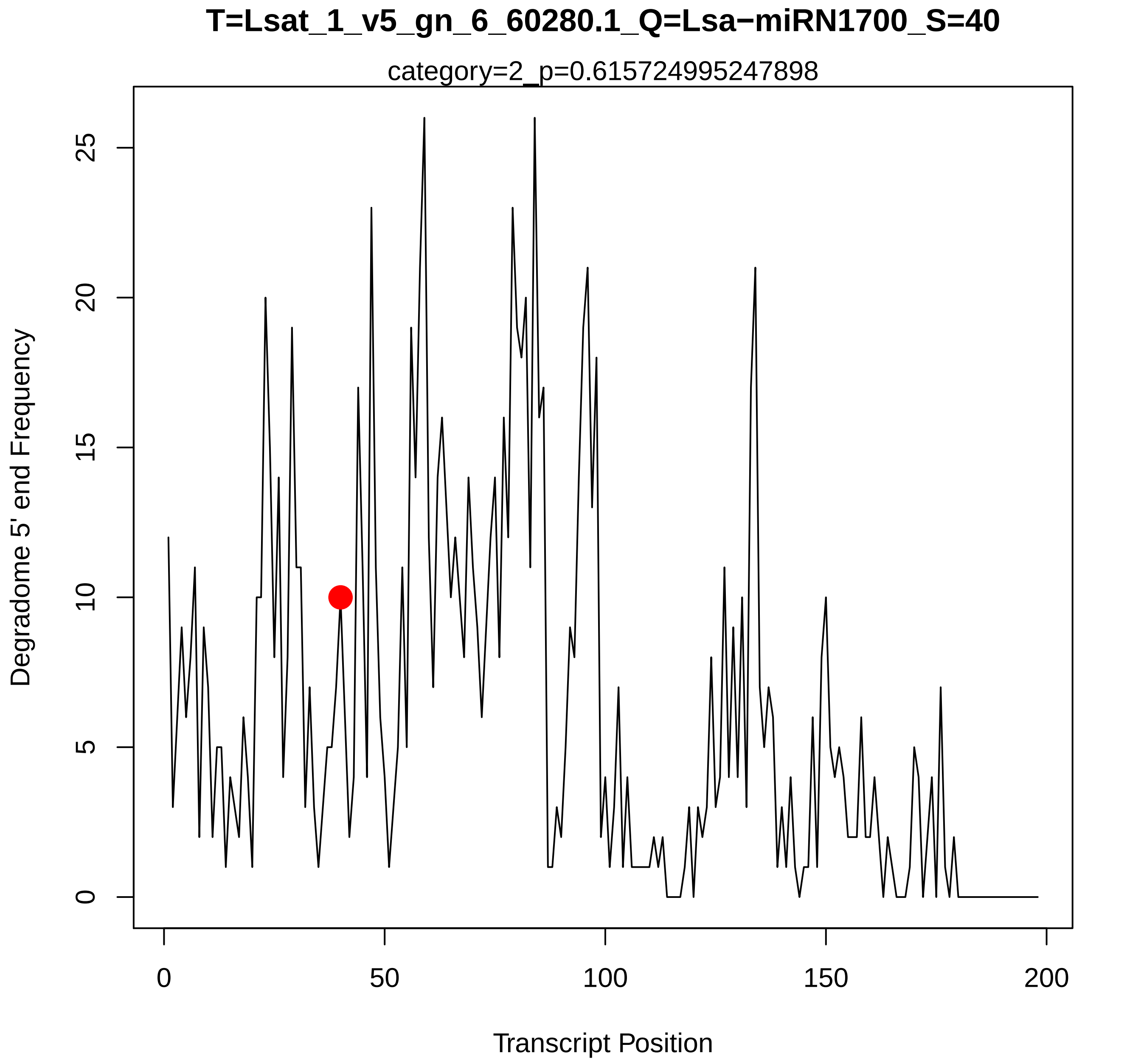

T=Lsat_1_v5_gn_6_60280.1_Q=Lsa−miRN1700_S=40
category=2_p=0.615724995247898
Degradome 5' end Frequency
Transcript Position
0
5
10
15
20
25
0
50
100
150
200
